# Comparison of the Efficacy and Safety of Aspirin and Low-Molecular-Weight Heparin in Patients With a Fracture: A Meta-Analysis

**DOI:** 10.7759/cureus.39025

**Published:** 2023-05-15

**Authors:** FNU Nimerta, Sana Faisal, Nafisa Reyaz, Syeda Urooba Shah, Swathi Gurajala, Raja Ram Khenhrani, Muhammad Waqas Khan, Adil Amin

**Affiliations:** 1 Medical College, Jinnah Sindh Medical University, Karachi, PAK; 2 Internal Medicine, California Institute of Behavioral Neurosciences & Psychology, California, USA; 3 Internal Medicine, Jinnah Sindh Medical University, Karachi, PAK; 4 Internal Medicine, Jawaharlal Nehru Medical College and Hospital, Aligarh, IND; 5 Medical Microbiology, College of Applied Medical Sciences, Imam Abdul Rahman Bin Faisal University, Jubail, SAU; 6 Internal Medicine, Liaquat University of Medical and Health Sciences, Karachi, PAK; 7 Internal Medicine, Services Institute of Medical Sciences, Lahore, PAK; 8 Cardiology, PNS (Pakistan Navy Ship) Shifa, Karachi, PAK

**Keywords:** low molecular weight heparin, aspirin, meta-analysis, fracture, venous thromboembolism

## Abstract

The aim of this study is to compare the efficacy and safety of aspirin and low-molecular-weight heparin (LMWH) in preventing thromboembolic events in patients with fractures. The present meta-analysis was reported according to the Preferred Reporting Items for Systematic Reviews and Meta-Analyses (PRISMA) guidelines. We searched EMBASE, PubMed, and EBSCO to find articles comparing aspirin and LMWH in patients with orthopedic trauma from inception to April 15, 2023. Limits were set to studies published in the English language only. Outcomes assessed in this meta-analysis included VTE and all-cause mortality. VTE can manifest as deep venous thrombosis (DVT) and pulmonary embolism. For safety analysis, rates of wound complication, infection, and bleeding complications were compared between the two study groups. A total of three studies were included in this meta-analysis enrolling 12884 patients. The study found no significant difference between the two groups in the risk of DVT and pulmonary embolism, and aspirin was non-inferior to LMWH for the prevention of all-cause mortality in patients. Additionally, no significant safety risk was associated with aspirin thromboprophylaxis. These findings suggest that inexpensive over-the-counter aspirin is as effective as LMWH in terms of safety and efficacy profile, making it a feasible option to consider in clinical practice.

## Introduction and background

Venous thromboembolism (VTE) is a potentially fatal complication that can occur after orthopedic trauma [1-2]. Patients who are hospitalized after a fracture are at increased risk of VTE due to the traumatic injury itself, limited mobility during the recovery phase, and the duration of surgery [3]. Several clinical guidelines recommend the use of thromboprophylaxis therapy to reduce the risk of complications and death associated with VTE after orthopedic injuries [4-5]. There has been considerable discussion regarding which thromboprophylaxis treatment is best, taking into account the costs and risks of bleeding associated with each option. There are different anticoagulant therapies available for VTE prophylaxis, including newer oral agents and injectable agents such as low-molecular-weight heparin (LMWH) [6].

Considerable evidence suggests that platelet activation and the recruitment of neutrophils are involved in the onset and spread of deep vein thrombosis (DVT) [7]. Therefore, it is logical to assume that the use of low-dose aspirin may have a preventive impact on VTE by decreasing platelet activation and, to some degree, through the acetylation of proteins that participate in blood coagulation such as fibrinogen and prothrombin. This acetylation results in more effective fibrinolysis and reduced thrombin production [8].

The Pulmonary Embolism Prevention (PEP) trial clearly demonstrated that low-dose aspirin reduces the risk of pulmonary embolism (PE) and DVT by at least one-third in patients undergoing hip fracture surgery or elective arthroplasty when compared to placebo during a period of increased risk [9]. Recent trials and meta-analyses have discovered that aspirin could be a viable alternative to LMWH for thromboprophylaxis in patients who have undergone total joint arthroplasty, with a safer profile [10-11]. Despite this, anticoagulant thromboprophylaxis remains the preferred method, and aspirin is currently used off-label for the prevention of VTE in both the USA and Europe [6].

Evidence from head-to-head comparisons among patients with fractures who have been treated operatively is limited. If the clinical outcomes of thromboprophylaxis options are comparable, patients with fractures tend to prefer aspirin due to its lower cost and oral administration compared to the subcutaneous injection of LMWH [1]. This meta-analysis aims to combine the results of published literature to compare the efficacy and safety of aspirin and LMWH in preventing thromboembolic events in patients with fractures.

## Review

Methodology

The present meta-analysis was reported according to the Preferred Reporting Items for Systematic Reviews and Meta-Analyses (PRISMA) guidelines. We searched EMBASE, PubMed, and EBSCO to find articles comparing aspirin and LMWH in patients with orthopedic trauma from inception to April 15, 2023. Limits were set to studies published in the English language only. The search terms used to carry out the search included "aspirin," "venous thromboembolism," "low-molecular-weight heparin," "fracture," and "orthopedic trauma," along with medical subject headings (MeSH) terms. These terms were combined using Boolean algebra operators. Additionally, the reference list of all included studies was manually searched. The search was carried out by two authors independently, and any disagreement between the two authors was resolved through discussion.

Selection Criteria

The following criteria were required for the included studies: (a) compared aspirin and LMWH in patients with fractures; (b) studies conducted in adults only; and (c) published in the English language. Studies that did not report required outcomes were excluded. Literature reviews, animal experiments, laboratory studies, and case reports were also excluded.

Data Extraction, Outcomes, and Quality Assessment

Data were extracted from included studies using a pre-designed data extraction form developed on Microsoft Excel. Data extraction from studies included author name, year of publication, sample size, dose of drugs, follow-up duration, sample size, and participant characteristics. Outcomes assessed in this meta-analysis included VTE and all-cause mortality. VTE can manifest as deep venous thrombosis (DVT) and pulmonary embolism. For safety analysis, rates of wound complication, infection, and bleeding complications were compared between the two study groups.

The Cochrane Risk of Bias criteria were used for the quality assessment of randomized control trials (RCTs). A total of seven items were used to assess the risk of bias in each study. Each quality item was graded as low risk, high risk, or no clear risk. For observational studies, quality assessment was done using the Newcastle-Ottawa scale.

Statistical Analysis

The data analysis was conducted using RevMan 5.3 software (Review Manager (RevMan) (Computer program). Version 5.3, The Cochrane Collaboration, 2020). For counting data, the effect size used was the Relative Risk (RR), and the results were presented with each effect size and its corresponding 95% confidence interval (CI). Clinical heterogeneity was tested for the included studies, and if the heterogeneity was less than 50% (I2<50%), the fixed-effect model was selected for meta-analysis. However, if the heterogeneity was greater than 50% (I2>50%), it indicated heterogeneity among the studies, and a random-effect model was used.

Results

**Figure 1:**
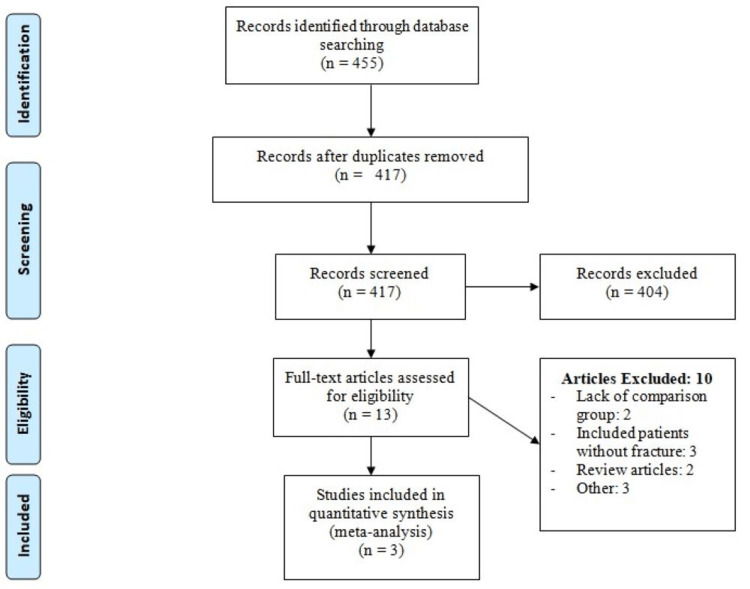
Process of study selection

Figure 1 shows the process of study selection. Online database searching yielded 455 related studies. After reviewing titles and abstracts, 417 unrelated studies were excluded and the full text of 13 studies was obtained. Through a detailed assessment of 13 full-text studies, three studies fulfilled predefined inclusion criteria and were included in the present meta-analysis with a total of 12884 patients. Table 1 shows the characteristics of the included studies. Out of the three included studies, two were RCTs while one was an observational study. Table 2 shows the quality assessment of the included studies.

**Table 1:** Characteristics of included studies RCT: randomized control trial; VTE: venous thromboembolism; NR: not reported

Author Name	Year	Study Design	Participants	Groups	Sample Size	Follow-up	Age (Years)	VTE History (%)
Chisari et al [12]	2022	Retrospective	Patients with a femoral neck fracture undergoing hip arthroplasty	Aspirin	172	3 Months	74.9 vs 78.9	0 VS 0
				Control	172			
Haac et al [13]	2020	RCT	All adult trauma patients admitted with an operative extremity fracture proximal to the carpals or metatarsals or any hip or acetabular fracture requiring VTE prophylaxis	Aspirin	165	3 Months	48.0 vs 45.4	4.8 vs 4.3
				Control	164			
METRC [14]	2023	RCT	fracture of an extremity (anywhere from the hip to midfoot or shoulder to wrist)	Aspirin	6101	3 Months	44.5 vs 44.7	NR
				Control	6110			

**Table 2:** Quality assessment

Quality Assessment for Randomized control trial						
Study ID	Selection Bias	Performance	Attrition	Reporting	Other	Overall
Haac et al [13]	Low	High	Low	Unclear	Low	Moderate
METRC [14]	Low	High	Low	Low	Low	Moderate
Quality Assessment for Retrospective Studies						
Study ID	Selection	Comparability	Outcome	Overall		
Chisari et al [12]	3	2	2	High		

Pulmonary Embolism and DVT

**Figure 2:**
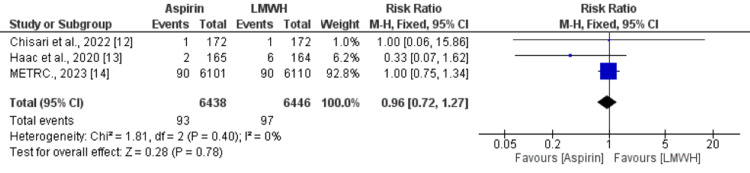
Effect of aspirin on pulmonary embolism Sources: References [12-14]

**Figure 3:**
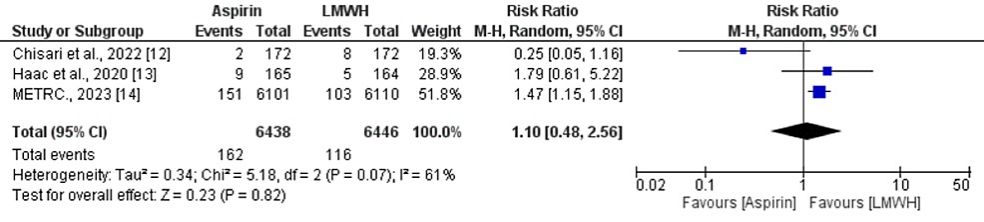
Effect of aspirin on DVT Sources: References [12-14] DVT: deep venous thrombosis

Three studies assessed pulmonary embolism and DVT. In terms of incidence of pulmonary embolism, no significant difference is reported between patients who received aspirin (1.44%) and patients who received LMWH (1.50%) (RR: 0.96, 95% CI: 0.72-1.27) as shown in Figure 2. No significant heterogeneity was reported among the study results (I-square: 0, p-value: 0.40). In relation to the incidence of DVT, a pooled analysis showed no difference between the two study groups (RR: 1.10, 95% CI: 0.48-2.56) as shown in Figure 3. Significant heterogeneity was reported among the study results (I-square: 61%, p-value: 0.07).

All-Cause Mortality

**Figure 4:**
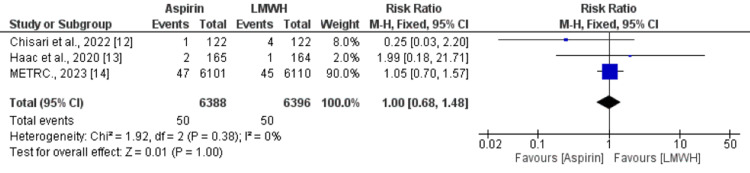
Effect of aspirin on all-cause mortality Sources: References [12-14]

Three studies compared all-cause mortality between patients who received aspirin and patients who received LMWH. The pooled rate of mortality was 0.78% and no significant difference is there between the two groups (RR: 1.00, 95% CI: 0.68-1.48) as shown in Figure 4. No significant heterogeneity was reported among the study results (I-square: 0, p-value: 0.38).

Safety Analysis

For safety analysis, we assessed the rate of bleeding complications, wound complications, and infections. No significant differences were noted in any of the safety outcomes between patients who received aspirin and patients who received LMWH as shown in Table 3. No significant heterogeneity was reported among the study results in any of the safety outcomes.

**Table 3:** Results of the safety analysis RR: risk ratio; CI: confidence interval

Outcome	Number of Studies	RR (95% CI)	I-square
Bleeding Complications	2	0.96 (0.89-1.05)	0%
Wound Complications	2	0.78 (0.39-1.56)	35%
Infections	2	1.10 (0.84-1.44)	0%

Discussion

Venous thromboembolism is an acute and potentially life-threatening disease with a risk of recurrence [15]. VTE can manifest as DVT and pulmonary embolism. The present meta-analysis compares the efficacy of aspirin with LMWH in preventing VTE in patients with fractures. The study found no significant difference between the two groups in the risk of DVT and pulmonary embolism. We also found that aspirin was non-inferior to LMWH for the prevention of all-cause mortality in patients. Additionally, no significant safety risk was associated with aspirin thromboprophylaxis.

Previous studies have reported a higher incidence of VTE in patients who have had orthopedic trauma or undergone major orthopedic surgery. For instance, Imberti et al. conducted a study including 69,770 patients and found that at least 2393 patients developed VTE during follow-up [16]. Following ankle and foot surgery, Huntley et al. examined 23,212 patients and found an incidence of VTE of 0.6% [17]. Basques et al. assessed 4412 patients undergoing surgery for ankle fractures and found the incidence of VTE to be 0.8% [18]. Considering the risk of VTE in this population, thromboprophylaxis is an essential part of the treatment plan. Given the risk of venous thromboembolism (VTE) in this particular population, the inclusion of thromboprophylaxis as a crucial element in the overall treatment plan is essential.

This is the first meta-analysis comparing aspirin with LMWH in patients with fractures. Previous studies have focused on patients with elective hip and knee replacement. Our observation reveals insignificant differences regarding DVT. Previous studies that included patients who underwent knee or hip arthroplasty have reported similar findings [19-20]. The Pulmonary Embolism Prevention (PEP) trial suggested that aspirin decreases the risk of DVT and pulmonary embolism by at least a third throughout a period of increased risk. As a result, compelling evidence is now available to support the routine use of aspirin in various surgical and medical populations that face a high risk of developing venous thromboembolism [9].

After discovering that both daily aspirin and injections offer similar outcomes in preventing serious blood clot-related outcomes in patients with fractures, many may prefer taking the former. Studies demonstrate that inexpensive over-the-counter aspirin is as effective as LMWH in terms of safety and efficacy profile, making it a feasible option to consider in clinical practice. However, considering that only three studies were included in the present meta-analysis, these findings need to be interpreted with caution. More prospective RCTs are required to evaluate the benefits of aspirin in patients with fractures.

Each year, an estimated one million Americans are hospitalized due to extremity fractures. This new discovery could assist in preventing potentially life-threatening blood clots in such patients by administering a cheaper and more convenient medication. If a healthcare professional recommends the use of low-dose aspirin for preventing cardiovascular disease, nine out of 10 adults are likely to comply with the recommendation. This indicates that patients widely acknowledge the effectiveness of the medication as a treatment regimen [21].

The current meta-analysis has certain limitations. First, only three studies were included in the present meta-analysis, and due to the limited availability of data, we were not able to perform a subgroup analysis. Second, considering the variability in the clinical guidelines for the duration of thromboprophylaxis at different healthcare institutions, we did not mandate the thromboprophylaxis duration. However, differences in the thromboprophylaxis therapy duration after hospital discharge may have affected the outcome. Notwithstanding these limitations, our results hold clinical significance. Patients who have experienced orthopedic trauma prefer aspirin over low-molecular-weight heparin because of the former's affordability and convenience in administration [22]. In hospitalized patients, oral medications are less likely to be omitted as compared to injectable thromboprophylaxis drugs. However, further trials are required to warrant these findings and to develop clinical recommendations on the use of aspirin in patients with fractures.

## Conclusions

In conclusion, this meta-analysis compared the efficacy of aspirin with LMWH in preventing VTE in patients with fractures. The study found no significant difference between the two groups in the risk of DVT and pulmonary embolism, and aspirin was non-inferior to LMWH for the prevention of all-cause mortality in patients. Additionally, no significant safety risk was associated with aspirin thromboprophylaxis. These findings suggest that inexpensive over-the-counter aspirin is as effective as LMWH in terms of safety and efficacy profile, making it a feasible option to consider in clinical practice. However, the limitations of this study, such as only three studies being included, mean that further research is needed to evaluate the benefits of aspirin in patients with fractures. Overall, the use of aspirin could help in preventing potentially life-threatening blood clots in patients with fractures by administering a cheaper and more convenient medication.
